# Pooled Enrichment Sequencing Identifies Diversity and Evolutionary Pressures at NLR Resistance Genes within a Wild Tomato Population

**DOI:** 10.1093/gbe/evw094

**Published:** 2016-04-27

**Authors:** Remco Stam, Daniela Scheikl, Aurélien Tellier

**Affiliations:** Section of Population Genetics, Technische Universität München, Freising, Germany

**Keywords:** resistance genes, population genetics, RENSeq, *Solanum penellii*

## Abstract

Nod-like receptors (NLRs) are nucleotide-binding domain and leucine-rich repeats containing proteins that are important in plant resistance signaling. Many of the known pathogen resistance (R) genes in plants are NLRs and they can recognize pathogen molecules directly or indirectly. As such, divergence and copy number variants at these genes are found to be high between species. Within populations, positive and balancing selection are to be expected if plants coevolve with their pathogens. In order to understand the complexity of R-gene coevolution in wild nonmodel species, it is necessary to identify the full range of NLRs and infer their evolutionary history. Here we investigate and reveal polymorphism occurring at 220 NLR genes within one population of the partially selfing wild tomato species *Solanum pennellii.* We use a combination of enrichment sequencing and pooling ten individuals, to specifically sequence NLR genes in a resource and cost-effective manner. We focus on the effects which different mapping and single nucleotide polymorphism calling software and settings have on calling polymorphisms in customized pooled samples. Our results are accurately verified using Sanger sequencing of polymorphic gene fragments. Our results indicate that some NLRs, namely 13 out of 220, have maintained polymorphism within our *S. pennellii* population. These genes show a wide range of π_N_*/*π*_S_* ratios and differing site frequency spectra. We compare our observed rate of heterozygosity with expectations for this selfing and bottlenecked population. We conclude that our method enables us to pinpoint NLR genes which have experienced natural selection in their habitat.

## Introduction

Resistance genes are important players in the interaction between plants and pathogens. They are involved in direct and indirect recognition of effector molecules from the pathogen and are hence thought to be under constant evolutionary pressure.

Most resistance genes (hereafter R-genes) including the best characterized ones belong to the NLR (nod-like receptors) or NB-LRR (nucleotide-binding site and leucine-rich repeat containing) type (Caplan et al. 2008). These include important R-genes from many food crops like Bs2 in pepper (Tai et al. 1999), R3a in potato (Huang et al. 2004), and Mi in tomato (Rossi et al. 1998). All NLR genes code for receptor proteins with a nucleotide-binding site (NB) and C-terminal leucine-rich repeats (LRR). Generally, these NB-LRRs can be divided into two groups, based on the sequence of their NB-ARC domain (a nucleotide-binding adaptor shared by APAF-1, certain *R-*gene products, and CED-4) and their N-terminal domains. One group has N-terminal domains related to the toll and interleukin receptors (TIR) and is also called TNL, whereas the second non-TIR group often contains a coiled coil (CC) and is also referred to as CNL (McHale et al. 2006).

Resistance conferred by R-genes was thought to predominantly come from direct gene-for-gene interaction between the R-gene and pathogen avirulence effectors (Flor 1971). This recognition results in a strong defense response, called effector triggered immunity, which in place results in the production of reactive oxygen species or a hypersensitive response in the plant. This reaction leads to localized cell death and thus stops the spread of the pathogen (Morel and Dangl 1997). Several indirect modes of action have also been described. In these cases, NLRs detect the modification of a (guarded) target protein which triggers a similar defense response (Van der Biezen and Jones 1998; McHale et al. 2006). Several examples exist that confirm direct interactions (Dodds et al. 2006), even though few sites for direct interaction are known.

R-gene effector interaction might also be more complex. In wheat, Lr10 and RGA, both NLRs, need to be present simultaneously to confer leaf rust resistance (Loutre et al. 2009). In tomato, NRC proteins are required for resistance conferred by several other NLR (Gabriels et al. 2007; Wu et al. 2016). When overexpressed in planta, individual domains of Rx, a tobacco virus NLR, interact with each other (Moffett et al. 2002). In rice, multiple NLRs and their various combinations have been linked to highly redundant resistance profiles (Zhang et al. 2015).

The effector–NLR interactions are crucial to determine the outcome of infection. NLRs are therefore expected to show variations and evidence of selective pressures. In this light, NLRs are often found as large gene families and consequently annotation, origin, and evolution of NLRs in plants (and animals) are an important field of study (Maekawa et al. 2011; Jacob et al. 2013). The numbers of identified NLR differ greatly within and between plant families, but also based on annotation methods (Jupe et al. 2013). In *Arabidopsis thaliana*, about 150 NLR genes have been identified (Meyers et al. 2003). In Solaneceous species like tomato and potato this number rises to about 355 and 438, respectively (Jupe et al. 2012; Andolfo et al. 2014). In rice so far 466 NLRs have been annotated (Li et al. 2010). No clear correlations seem to exist among age, genome size, and number of NLR because, for example, in the brassica family *Brassica rapa*, which has a similar sized genome to *A. thaliana*, has only 80 known NLRs (Mun et al. 2009).

In *A. thaliana*, NLR genes are located clusterwise on the genome and due to their hypervariable nature a model of a rapid birth and death process was suggested to explain expansion and diversification of the gene family (Michelmore and Meyers 1998). The 150 NLRs identified in *A. thaliana* are very divergent, but it is possible to cluster many of them together in groups by sequence similarity, while some remain orphan. Of the 22 groups, 10 groups show genes with positively selected positions. The number of sites however varies from 1 to 26; while the majority of selected sites occur in the LRR region, still 33 out of 116 are located in the NBS domain or other regions (Mondragón-Palomino et al. 2002). Studies of worldwide within-species variability of NLRs demonstrated the strong pervasive selection pressure. NLRs are thus likely to evolve under neutrality or purifying selection, and few under balancing selection (Stahl et al. 1999; Bakker et al. 2006). A study including sequence data from both *A. thaliana* and *Arabidopsis lyrata* showed similar results using divergence estimates, and indicated that the genes unique to a species, for example, lacking homologs, appeared to show weaker selective pressure and less copy number variation (CNV; Guo et al. 2011).

Other studies focused on comparing the NLR complement between multiple species, and 2,363 NLRs were identified in 12 eudicot plants, including 6 crop species. Of these genes, 50% show tandem duplications associated with strong positive selection (the ratio of nonsynonymous to synonymous substitutions, *K*_a_/*K*_s_ > 1.5). However, a small set of NLRs appears to be conserved for over 100 Myr in most eudicot genomes (Hofberger et al. 2014). In monocots, the divergence between species appears to be large, as numbers of NLRs differ greatly among maize, sorghum, brachypodium, and rice (Li et al. 2010). NLR clusters built from phylogenetic methods can exhibit a wide range of *K*_a_/*K*_s_ ratios (0.5–3.3) (Yang et al. 2013). Because between-species comparisons have lower statistical power to detect selection if divergence is high (Gharib and Robinson-Rechavi 2013), and they do not allow detecting the occurrence of balancing selection, we investigate within-population variation to understand short-term evolution of NLRs.

Wild Solanum species provide the optimal model organisms for such studies. During its domestication *Solanum lycopersicum* has suffered significantly from a reduction in genetic diversity (100 Tomato Genome Sequencing Consortium et al. 2014; Lin et al. 2014). Hence, wild tomato species regularly serve as germplasm source in current breeding programs, making them economically interesting to study (Bai and Lindhout 2007). In addition, genomic resources are already available for a selection of wild and cultivated tomato.

In this study, we make use of *Solanum pennellii.* This wild species contains several disease-resistance loci, including canonical NLRs, against Oomycete pathogen *Phytophthora infestans* (Smart et al. 2007). It is the source for the *I-1* and *I-3* genes which confer resistance against Fusarium wilt (Sarfatti et al. 1991; Scott et al. 2004). It also contains other resistance loci, like *RXopJ4*, a bacterial spot resistance locus (Sharlach et al. 2012), and thus has large value for plant breeders. *Solanum pennellii* LA0716 has been used to develop introgression lines with *S. lycopersicum* cultivar M82, which has been instrumental in understanding yield parameters and generating increased yields (Eshed and Zamir 1994; Eshed et al. 1996; Gur and Zamir 2004). *Solanum pennelli* is a self-compatible species which is expected to show low levels of within-population diversity. The recent sequencing of one plant of *S. pennellii* LA0716 yielded a high quality reference genome and led to the identification of a number of abiotic stress associated genes (Bolger, Scossa, et al. 2014).

The costs of generating NGS data are constantly dropping; however, for complex plant species with large genomes, sequencing costs and also computation time for mapping or assembly are still considerable. R-gene enrichment sequencing (RENSeq) can be used to reduce the complexity of the DNA sample, by enriching the R-gene component and thus reducing overall sequence complexity before sample submission. To this purpose, RENSeq has successfully been used to identify the NLR complement of both cultivated tomato and potato (Jupe et al. 2013; Andolfo et al. 2014). Nevertheless, for population genetic studies, ideally large numbers of individuals per population as well as large numbers of populations are desired to allow inference of short time-scale selective pressures, and thus driving up in return the sequencing costs. Recently, several studies have shown that pooled sequencing can dramatically reduce the sequencing costs, as well as time and costs associated with sample preparation (Schlötterer et al. 2014). Note that with pooled sequencing it is not possible to assign sequences to a single individual, but population genetics statistics can be successfully computed (Ferretti et al. 2013) and sampling uncertainties can be accounted for (Kofler et al. 2011; Lynch et al. 2014). Pooled sequencing has been successfully used to study population evolution in, for example, quail (Boitard et al. 2013), drosophila (Zhu et al. 2012), arabidopsis (Fracassetti et al. 2015), and the wild tomato species *Solanum chilense* (Böndel et al. 2015). Here we show proof of principle that pooled RENSeq can be used to identify R-genes of interest within a single population.

Our overall aim is to identify R-genes that maintain polymorphisms within wild populations. As a first step, we provide proof-of-principle in *S. pennellii.* Due to its limited genetic diversity, *S. pennellii* is particularly suited to test the statistical power of various population genetics methods on pooled data. We accurately identify a large set of NLR genes in the species and provide robust analysis to identify single nucleotide polymorphisms (SNPs) and calculate population genetics statistics. With this, we show that a small subset of R-genes maintains particular high diversity within *S. pennellii.*

## Methods

### NLR Identification, Analysis, and Probe Design

To identify high confidence NLR genes, we used the published *S. pennelli* sequence data and NLRParser as recommended by the authors (Steuernagel et al. 2015) We ran MAST (Bailey et al. 2009) (1 × 10^−6^) using previously described NLR-associated motifs (Jupe et al. 2012). Matching sequences were extracted and submitted to NLRParser for annotation. The output was used to extract gene sequences and gff files with predicted protein annotations, to be used in follow-up analysis. A phylogenetic tree based on protein alignment was constructed using the extracted NB-ARC domains of the identified NLR. All domains were aligned with MUSCLE (Edgar 2004). Manual curation and removal of the biggest gaps was done in jalview (Waterhouse et al. 2009) before construction of the tree with PhyML (Guindon et al. 2010) (WAG model, BioNJ starting tree, and NNI tree searching, 100 bootstraps).

Probes (supplementary file S2, Supplementary Material online) were designed using Agilent’s SureSelect Software with the predicted NLR for *S. pennellii* and published NLR for *S. lycopersicum*, *Solanum tuberosum*, and *A. thaliana*. We also included a set of 22 control genes used in previous evolutionary studies of potato or tomato (supplementary file S3, Supplementary Material online). These included five resistance signaling associated genes (Pto, Fen, Rin4, Prf, and Pfi) (Rose et al. 2011), three proteases (Rcr3, C14, and PIP1), and 14 metabolism-related genes, the so-called reference genes in Böndel et al. (2015). We used BLAST and a second run of NLRParser to confirm that all targeted sequences were indeed putative NLR genes. Several probes gave false positive hits (targeting LRR-containing, but non NLR genes). Those probes were manually removed. In total, 12,331 probes were selected to use with the SureSelect platform.

### Plants, DNA Extraction, and RENSeq

Ten *S. pennellii* plants (LA0716) were grown in our glasshouse under 16 h light conditions and a minimum temperature of 18 °C. The seeds were obtained from Wageningen University Centre for Genetic resources of the Netherlands (CGN). Leaf tissue was collected from 8-week-old plants and ground in liquid nitrogen. DNA was extracted using a CTAB (hexadecyl trimethyl–ammonium bromide) buffer based method (https://www.protocols.io/view/DNA-extraction-from-plants-eusbewe, last accessed May 3, 2016). The DNA was quantified using Life Technologies’ Qubit and quality confirmed with Agilent Bioanalyzer 2100. DNA for ten plants was pooled and NLR enrichment was performed according to Agilents SureSelect XT protocol with minor modifications: DNA was sheared on a Covaris S220 to 800 bp, size selection and cleaning was done using AMPure XP beads (Beckman Coulter) in two steps using 1.9:1 and 3.6:2 fragment DNA to beads ratio. The quality was assessed using a Bioanalyzer 2100 (Agilent). End repair, adenylation, and adaptor ligation were performed as described by Agilent. Precapture amplification was done using Q5 high-fidelity PCR mixes. The amplified library was quality checked on a Bioanalyzer 2100. Hybridization was performed as suggested for libraries <3 Mb. The library was indexed with 8-bp index primers using Q5 PCR mix and quality was assessed using the Bioanalyzer 2100 and quantified using Qubit. Our library was pooled with seven other samples in equal DNA amounts and the resulting pool was quantified by qPCR using the NGSLibrary quantification kit for Illumina (Quanta biosciences) and diluted down to a final concentration of 20 nM. Illumina MiSeq was run twice on the same library following the manufacturer’s instructions for MiSeq v3. chemistry.

### Data Analysis

Our SNP detection methods are outlined in detail in supplementary figure S1, Supplementary Material online. FASTA files with sequencing data were quality controlled (QC) using trimmomatic (Bolger, Lohse, et al. 2014) (HEADCROP:3 SLIDINGWINDOW:4:30 TRAILING:30 MINLEN:40) and mapping was performed with trimmed reads using Stampy (Lunter and Goodson 2011) and BWA (Li and Durbin 2009) (default settings). Supplementary figure S2, Supplementary Material online, shows the quality scores before and after trimming. Low-quality mappings and duplicated reads were removed using Picard Tools (http://broadinstitute.github.io/picard/), before SNP calling. SNP calling was performed using Popoolation (Kofler et al. 2011), using the author’s recommended settings, min-cov was varied from 3 to 9 (supplementary fig. S3*A*, Supplementary Material online), and the expected allele count set to 20. We tried several subsampling methods. Supplementary figure S3B, Supplementary Material online, shows that subsampling in general appears to reduce the number of called SNPs and does not improve the quality. In addition, we used GATK Haplotypecaller and SelectVariants (McKenna et al. 2010). GATK allows for advanced filtering options, hence we used filters based on our Sanger sequenced data. We aligned our Sanger reads with the GATK data and manually optimized the values for all filters based on these sequenced regions. The used filters are outlined in supplementary file S5, Supplementary Material online. For completeness we used two more popular SNP callers Varscan2 (Koboldt et al. 2012) and BCFTools (http://www.htslib.org/) using default settings for polyploid organisms.

The classic population genetics statistic π (Tajima 1983) was computed based on the estimated minor allele frequencies using SNPGenie (Nelson et al. 2015). The folded site frequency spectrum (SFS) estimations were done using several methods. Pool-HMM (Boitard et al. 2013) was run to calculate the allele frequency in our data (option -spectrum) directly from the alignment file. These data were fed back into Pool-HMM (option -estim) to estimate absolute allele frequency and summarized into folded spectrum. Second, an SFS was calculated from GATK output (generated using HaplotypeCaller with -ploidy 20), by parsing expected allele frequencies from the filtered output VCF, folding and summarizing them. Finally, we used filtered Popoolation outputs and deduced SFS from the observed allele frequencies. We computed the ratio of nonsynonymous to synonymous diversity πN/πs using SNPGenie which uses an estimator based on the method of Nei and Gojobori (1986). Possible homologs for all the SNP containing genes were identified using BLAST against the curated swissprot database, to allow identification of homologs of evidence-based NLR. Only NLR with >30% sequence identity and over 70% coverage with the original NLR were reported.

### Sanger Sequencing

Primers were designed to anneal around at least one exonic region of the following genes: Sopen02g021920, Sopen12g030570, Sopen11g028610, and Sopen12g032710 (supplementary file S6, Supplementary Material online). Genes were amplified from DNA extracted from each of the individual plants used in our pool with Q5 polymerase (NEB), using the manufacturer’s recommendations. Amplified gene fragments were purified (Qiaprep Qiagen) and sequenced directly, or ligated into the pENTR-TOPO2.1 vector (Life technologies) and transformed into *Escherichia coli* TOP10 cells. Positive colonies were selected and plasmid DNA was extracted using Qiagen Qiaprep.

To identify all SNPs at each gene segment, we sequenced at least two plasmids per plant. We used CodonCode Aligner (CodonCode Inc) to check the sequence quality and align the plasmid sequenced with the reference genes. Up to 21 SNPs were manually annotated for each gene section.

### Visualization

Visualization of reads, annotations, motifs, and SNPs was done using IGV (Thorvaldsdóttir et al. 2013). Mapped reads were shown on the reference sequence and bedtools was used to generate custom tracks for the different NLR motifs, gene annotations, and SNPs. Graphs were made in R (R Foundation for Statistical Computing, Vienna, Austria), using the package ggplot.

## Results

### *Solanum pennellii* Contains 220 High-Confidence NLRs

**Fig. 1.—:**
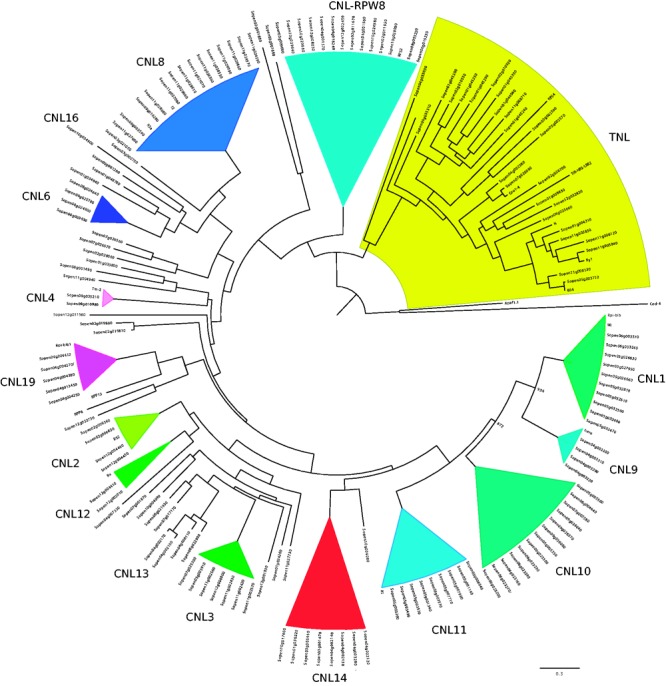
NLR genes in *Solanum pennellii*. Phylogenetic tree for the identified *S. pennellii* NLR genes generated using PhyML (WAG) with 1,000 bootstraps after alignment of all NB-ARC using MUSCLE. TNLs are highlighted in yellow background. Collapsed triangles represent known NLR clusters with high bootstrap values (>75%, clade CNL-RPW8: 54%). NLR families are indicated above the different clades and several named resistance genes from other species have been included for references.

The automated gene annotation for *S. pennellii* (Bolger, Scossa, et al. 2014) contains 486 proteins that contain domains associated with canonical NLRs. However, annotations are rather incomplete and describe only individual domains *(*214 NB-ARC; 259 LRRs; 13 CC, TIR, or other domains). As individual NB-ARC or LRR domains can also be part of other signaling proteins, like receptor-like proteases, careful reannotation was required. We reannotated *S. pennellii* proteins and inferred whether they were putative complete or partial NLRs, where complete NLRs contain an N-terminal region (CC or TIR), NB-ARC, and one or more LRRs. Partial genes lack one of the three domains. All partial genes are included in our analysis, because to date it is not known whether these are nonfunctional pseudogenes or whether some are functional R-genes. We ran NLRParser against the predicted proteins for *S. pennellii* V2. This yielded 220 putative NLRs, of which 93 were complete (supplementary file S1, Supplementary Material online). We found 164 members of the CNL class, 39 of the TNL class, 17 lacking their N-terminus. As in previous RENSeq studies (Jupe et al. 2013; Andolfo et al. 2014), manual inspection showed that some putative NLRs might be wrongly annotated in the *S. pennelli* V2 genome. Some of our reads aligned well outside the annotated genes. As we were not yet able to accurately predict coding regions lying within these reads, which will be required for calculation of population genetics statistics, these reads were ignored and we focused only on those NLRs for which coding region data were available. To show that our data set is likely to be a good representation of the NLRs to be found in *S. pennelli*, we constructed a phylogenetic tree based on the NB-ARC domain of the identified NLR. Figure 1 shows that our tree contains the main NLR classes that can be found in other tomato species and close homologs of known NLRs from unrelated species, similar to those described for *S. lycopersicum* and *Solanum pimpenellifolium.*

### Sequencing, QC, and Mapping Statistics

We used sequence data of the 220 predicted NLR together with previously annotated NLR from tomato (*S. Lycopersium*), potato (*S. tuberosum*), and previously described known NLR sequences (Jupe et al. 2012) to design NLR-specific probes (supplementary file S2, Supplementary Material online). DNA samples were sequenced as part of a larger pool. Two runs were done for our pool, which resulted in 805,122 and 2,147,039 reads. We performed basic quality control with Trimmomatic and trimmed all parts of the reads with quality lower than 30. Unpaired and low-quality read pairs were removed and finally we retained 669,869 and 1,283,203 high-quality paired reads. We were able to map 642,331 and 1,230,551 of the read pairs to the reference using Stampy for run1 and run2, respectively, and 494,012 and 986,210 read pairs using BWA. Downstream analysis revealed that the BWA alignment gave better results for the SNP calling, hence we thereafter report the values obtained with the BWA mapped reads only.

### RENSeq Provides Deep Coverage in Targeted Regions

**Fig. 2.—:**
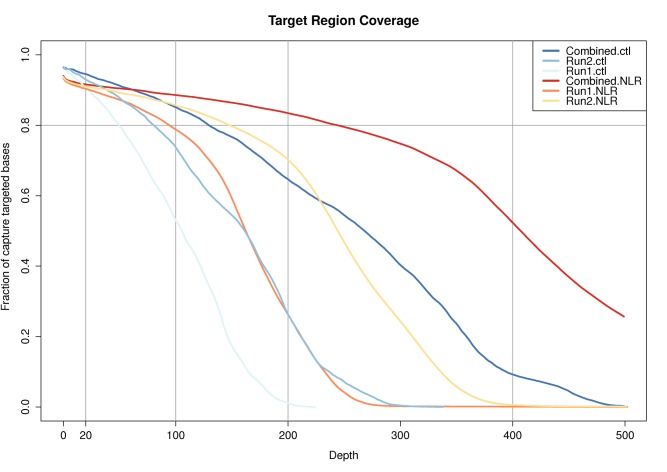
Coverage of targeted region. The fraction of bases in the targeted area having a coverage of a certain depth (*x*-axis) or deeper. The lines represent the individual runs and the combined data, separated for the NLR regions and the set of control (ctl) genes. The plot represents the data after preprocessing.

To assess the success of our enrichment sequencing, we plotted the depth of coverage per site against the fraction of the targeted region with the given coverage. Our probes were designed using exon data only, this reduces the coverage in intronic regions, but assures high read depth in coding regions. Figure 2 shows that close to 80% of the exonic target regions for the 22 control genes have a coverage of at least 130 reads, and 50% a coverage for at least 269 reads. For the NLRs, 80% of the predicted target region has a coverage of 245x or higher, and 50% of coverage of more than 408x. The difference in coverage between R-genes and control genes can be explained by the probe design. For R-genes, we have used a very redundant database containing all known tomato, potato, and arabidopsis R-genes and additional genes from other species, while, for the control genes, each gene was included only once. Hence an R-gene with orthologs in tomato, potato, and arabidopsis will have many more suitable probes in our probset than a control gene.

As our initial mapping might contain misaligned or duplicated reads and mapping over introns, we performed an additional series of quality controls and filtering as described in the Methods section before identification of SNPs in both control and NLR data sets. Figure 2 shows the coverage plot after deduplication and filtering. The coverage at the first quartiles (e.g., 75% of the regions have higher coverage) is 112×, 172×, and 251× in respectively run1, run2, and both runs combined, whereas the median coverage was 163×, 243×, and 346×, respectively. 

### GATK and Popoolation Show Highly Congruent SNP Calls in Our Population

Next we set out to identify SNPs in all exons of the NLR and control genes within our sequenced population. We ran Popoolation using different cut-off values to establish the maximum sensitivity while minimizing the number of false positives. SNPs were called for run1, run2, and both runs combined, with minimum coverage set at 20, 30, and 40. We assumed equal amounts of DNA per plant and an average coverage near 120 in the run with the lowest coverage, and we expect a singleton allele frequency of 1/20. Minor singleton alleles should thus be readily picked up in the majority of cases with a minimum SNP count of 5 or 6. supplementary figure S3, Supplementary Material online, shows that with low minor allele count (3–5) very large numbers of SNPs are detected, and that indeed after the count of 6 the detection curves flatten off. Importantly, differences between separate runs (and thus read depth) as well as the minimum overall depth tend to have a negligible effect on SNP calls (with mincount 5–9) (supplementary fig. S3, Supplementary Material online). However, at higher stringency we observe a loss of sensitivity (mincount > 10). To guarantee high-quality SNPs, we decided to keep the minimum depth for follow-up analysis at 30. This way, minor alleles occurring in frequency 4/20 can still be found with the minimum SNP count set at 6. Lowering the minimum count could increase false positive rates in highly covered regions due to possible PCR bias. We also calculated the average coverage for all exons of each predicted gene to assure no correlation between SNP and coverage. Subsampling strategies implemented by Popoolation appear to have detrimental effect on the SNP calling (supplementary fig. S3*B*, Supplementary Material online) and were not used. Using the setting described, in total 249 SNPs were identified in the NLRs.

Next we used GATK as a second method to verify the previously called SNPs by Popoolation. Using GATK we could predict 222 SNPs. We compared GATK predicted SNPs with our popoolation data. We found that 185 SNPs in 12 genes overlap between both data sets (table 1). We manually inspected all SNPs called uniquely for GATK and found that 20 were called because they showed difference from the reference genome but did not show polymorphism within the sample, 3 were called in low coverage (<30) regions, and 7 were called with GATK with fewer than 6 occurrences of the SNPs. The final six are close to indel regions. To avoid false SNP calling, we excluded those regions in Popoolation. We also analyzed all SNPs called only with Popoolation, and 28 appear to be on locations where also low-quality reads can be found and 4 are near too high coverage regions (likely PCR bias). We could not observe any oddities for the other 32.

**Table 1:** SNPs Identified in One Population of *Solanum pennellii* LA0716

Name	Popool	GATK	Both
Sopen01g033800	1	0	0
Sopen02g006820	0	2	0
Sopen04g002150	0	2	0
Sopen04g002170	0	1	0
Sopen04g003320	0	1	0
Sopen05g028830	0	2	0
Sopen05g032470	0	2	0
Sopen05g032480	5	2	2
Sopen05g032500	0	6	0
Sopen05g032510	8	6	4
Sopen06g003570	2	1	0
Sopen06g023160	6	5	5
Sopen06g023290	0	1	0
Sopen07g001870	0	1	0
Sopen07g017170	6	6	0
Sopen08g003220	0	1	0
Sopen09g023290	0	6	0
Sopen09g035210	2	0	0
Sopen10g024970	5	5	5
Sopen10g024980	0	1	0
Sopen11g027060	0	2	0
Sopen11g028330	24	14	14
Sopen11g028360	22	16	15
Sopen11g028600	0	1	0
Sopen11g028600	0	1	0
Sopen11g028610	41	22	21
Sopen12g022450	96	88	83
Sopen12g032710	10	19	10
Sopen12g032720	9	9	9
Sopen12g032730	10	10	10
Sopen12g032810	1	1	1
Sopen12g032830	1	0	0

**Fig. 3.—:**
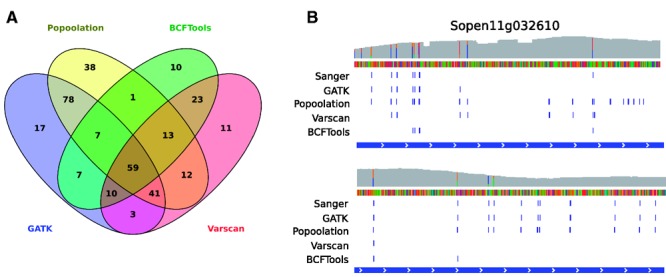
SNP calls from four different callers. (*A*) Overlap of called SNPs between different SNP callers. Popoolation and GATK share the most common SNPs. (*B*) SNPs called for a region of NLR Sopen11g028610. Top shows the coverage (gray) and SNPs that appear directly from the .bam file (including putative false positives). The blue lines in the lower parts of the figure show the SNPs as identified by Sanger sequencing and four SNP callers. Popoolation and GATK show the best performance judging by overlap.

We further tested Varscan and Bcftools to call SNPs in our data set; however, both these callers seem to underperform with 172 and 130 SNPs, respectively. Possible reasons might be that contrary to Popoolation and GATK, the versions we used have not been optimized for multiploid (>2) specimens or pooled data. Figure 3A shows a Venn diagram with the number SNPs called for each software. Popoolation and GATK together call the highest numbers of SNPs and also have the highest overlap.

We also used Popoolation and GATK to identify polymorphisms in our control genes. Overall, 12 SNPs were called in the control gene set by both tools, using settings previously described. One SNP was called by GATK only because it differed from the reference genome, but it did not show polymorphisms within our sample. Thus highlighting the importance of noting how SNP callers treat a reference sequence. As we are only interested in variation within our population (and not with the reference genome), such SNPs will be omitted in the remainder of this article.

### SNPs Can Be Verified Using Sanger Sequencing

To verify our SNP calling using Sanger sequencing, we designed primers annealing around one or more exons of two non-NLR genes, Sopen02g021920 (Rcr3) and Sopen12g030570 (C14), and two NLRs, Sopen11g028610 and Sopen12g032710 (supplementary file S3, Supplementary Material online). Our Sanger sequencing data confirm that Sopen02g021920 does not contain any polymorphisms (supplementary file S6, Supplementary Material online). For simple genomic regions, like those in Sopen12g030570 and Sopen12g032710, both GATK and Popoolation identified all Sanger sequenced SNPs. In complex regions, like part of Sopen11g028610, both GATK and Popoolation seem to call several, nonoverlapping false positive SNPs (fig. 3B). Due to its more flexible filtering we are better able to approach the true SNP set using GATK, yet no filtering method keeps in all positives and filters out all false negatives. Again, Varscan and BCFTools significantly underperform in this gene. To assure high-quality SNPs to calculate population genetics statistics, we will use SNPs as called by both GATK and Popoolation (table 1). This overlapping set shows lower false positive (3.6%) and false negative rates (6.4%) compared with the Sanger data than the individual SNP sets and also removes SNPs picked up because they only differ from the reference (see previous paragraph).

### Low Sequence Diversity Was Already Evident in the Original Population

Because we pick up low number of SNPs in our population, we wanted to infer how the maintenance of the plants in various collections affected genomic diversity in the NLRs. *Solanum pennellii* is a facultative selfing plant, and some loss of diversity can be expected. However, both the Tomato Genetic Resource Centre (TGRC, UC Davis, USA) and the Centre for Genetic Resources of the Netherlands (CGN, Wageningen University, the Netherlands), who maintained this population, confirm that since acquisition (by TGRC in 1958 and Wageningen from 1985) no more than 5–10 reproductive rounds have taken place and multiple plants were used in the process of multiplication. This reasoning is based on information provided by TGRC (Chetelat R, personal communication) and Wageningen University (Dooijeweert WV, personal communication). We can therefore reconstruct the following population model. We assume an initial heterozygosity *H*_0_ which is defined here as the probability to sample two alleles which are different in a population (Charlesworth and Charlesworth 2010) at the time of sampling. If one plant was initially sampled, the first generation of multiplication by selfing decreases heterozygosity by half to a value of *H*_1_ = 0.5*H*_0_. If two or more plants were sampled and crossed to produce F1, a proportion 0.5*s* of heterozygosity is lost due to the selfing rate *s*, yielding *H*_1_ = (1 − 0.5*s*)*H*_0_. Subsequently, between 8 and 12 diploid plants were produced every generation and crossed randomly in TGRC and CGN. In such randomly mixing population of size 2*N* = 16 or 2*N* = 24 chromosomes, the expectation for the decrease in heterozygosity between two consecutive generations (*t* and *t* + 1) is *H_t_*_+__1_ = (1 − 1/2*N*)*H_t_*. At the time point of our sample, the number of NLR genes showing heterozygosity is *H*_sample_ = 13/220. Applying these formulae, we can estimate the initial heterozygosity after *t* rounds of mutliplication as *H*_0_ = *H*_sample_/[*H*_1_(1−1/2*N*)*^t^*]. The initial proportion of heterozygote NLR loci in the initial wild population of *S. pennellii* would therefore be between *H*_0_ = [0.17, 0.21] for *s* = 1, and *H*_0_ = [0.12, 0.14] for *s* = 0.5, when assuming *t* = 10 generations of multiplication. For convenience, heterozygosity equates here with the proportion of polymorphic loci in our 220 NLRs with the population sample of 10 diploid plants (20 chromosomes). Increasing the number of initial plants would lower the expected initial heterozygosity even more. Hence, we can conclude that *S. pennellii* LA0716 must have had very low original diversity with more than 75% of the NLR showing no polymorphisms.

### Different Site Frequency Spectrum Estimators Yield Comparable Results

**Fig. 4.—:**
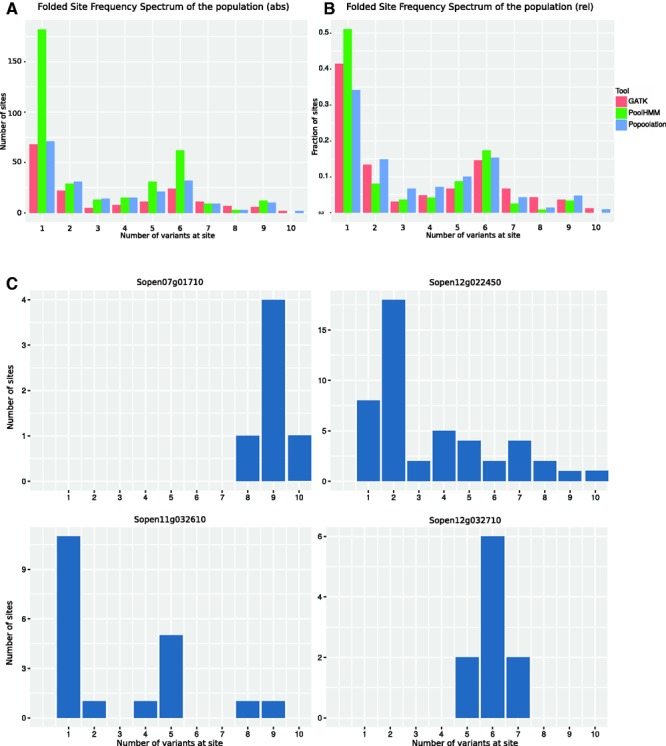
Site frequency spectra. Folded site frequency spectra for the SNPs detected in our NLR set. *x*-axis shows the number of variants per site, with ten equals a frequency of 0.5 in our population. (*A*) Absolute folded SFS; *y*-axis shows actual number of sites. (*B*) Relative folded SFS, *y*-axis shows the fraction of sites. (*C*) Absolute folded SFS per gene.

We used different methods to estimate the SFS of our NLR data. Pool-HMM (Boitard et al. 2013) calculates an allele frequency spectrum (SFS) directly from the mapped reads and uses this as a prior to estimate SNP frequency at a given location. We used GATK to infer allele frequency in HaplotypeCaller (using -ploidy 20), expected allele frequencies were then extracted after filtering. Finally, we estimated allele frequency from the Popoolation output data on minor alleles in our data set. All individual SNP frequencies were summed and turned into a folded SFS of the population. Figure 4A shows that in absolute values, Pool-HMM shows many more singletons and overall SNPs in the data, but this is likely due to the absence of the necessary filtering options. The relative SFS calculated from Pool-HMM and GATK derived data show very strong congruence (Pearson correlation = 0.98).

Interestingly, our folded SFS shows an increase for class 5–7. Inspection of SFS per gene reveals that, due to the low number of SNPs in our data, single genes with outlying SFS can be responsible for this pattern. Individual patterns for some R-genes show that indeed the genes seem to have differing spectra (fig. 4B). Sopen12g022450 shows an expected spectrum with high singleton count and flattening tail. Sopen07g01710 shows an increase in SNPs with intermediate frequency (greater than eight), whereas Sopen12g032710 shows an odd pattern with many SNPs occurring five to seven times, hence causing this intermediate frequency increase in the global SFS (fig. 4C).

### NLR Genes Show Differential Evolutionary Patterns

None of our 14 house-keeping control genes show any polymorphisms. For the pathogen-related control genes, only one out of eight (Sopen12g030570) had a significant number of SNPs within our population and a Ts/Tv ratio of 2.33. We identified 235 SNPs in our NLR data, with an average Ts/Tv ratio of 1.13. These SNPs were concentrated in only 13 NLRs. Strikingly, the numbers of SNPs per gene range from 1 to 66 and are not correlated to gene length or average coverage depth (*r* = 0.42 and 0.16). All genes meet the minimum coverage criteria in over 88%. Nucleotide diversity is measured within the population as π per site and per gene (table 2). Variation in π per gene ranges in two orders of magnitude between the different NLRs.

**Table 2:** Characteristics of Polymorphic NLR in *Solanum pennellii* LA0716

Gene	SNPs	Pi (SNPGenie)	Non_syn	Syn	PiN	PiS	PiN/PiS	Annotated	Homology	SNPs in
Sopen05g032480	2	0.00018	2	0	0.00023	0.00000	NaN	Complete	Unknown	NBARC
Sopen05g032510	4	0.00010	3	1	0.00011	0.00006	1.91586	Complete	Unknown	NBARC
Sopen06g023160	5	0.00091	5	0	0.00117	0.00000	NaN	Partial	R1A	NBARC
Sopen07g017170	6	0.00085	6	0	0.00109	0.00000	NaN	Partial	Unknown	All
Sopen10g024970	5	0.00062	4	1	0.00065	0.00053	1.20837	Partial	Unknown	Cterm
Sopen11g028330	14	0.00025	10	4	0.00022	0.00034	0.66187	Complete	RPP13-like	Nterm-Cterm
Sopen11g028360	15	0.00018	11	4	0.00016	0.00025	0.63918	Complete	RPP13-like	Nterm-Cterm
Sopen11g028610	21	0.00107	12	9	0.00093	0.00153	0.61044	Complete	RPP13-like	Nterm-Cterm
Sopen12g022450	83	0.00586	64	19	0.00538	0.00755	0.71278	Partial	Unknown	Cterm
Sopen12g032710	10	0.00370	5	5	0.00238	0.00852	0.27884	Partial	Unknown	Nterm-Cterm
Sopen12g032720	9	0.00338	6	3	0.00284	0.00536	0.53088	Partial	Unknown	Nterm-Cterm
Sopen12g032730	10	0.00149	3	7	0.00057	0.00475	0.11988	Complete	RPP8	NBARC-Ctem
Sopen12g032810	1	0.00051	0	1	0.00000	0.00241	0.00000	Partial	Unknown	Cterm

The assumptions that make *K*_a_/*K*_s_ ratio a reliable estimator for selective pressure on R-genes between species are not met when analyzing data within populations (Kryazhimskiy and Plotkin 2008). To assess potential selective pressures we calculated πN/π*s* for all R-genes, which is a better measure within populations (Charlesworth and Charlesworth 2010) (table 2). In our set, overall, partial NLR genes show higher values for π*N/*πs; however, many complete and partial NLR did not show any polymorphisms at all. Two NLRs (Sopen05g032510 and Sopen10g02490) show high (>1) π*N/*πs values and three others (Sopen05g032480, Sopen06g023160, Sopen07g017170) contain several nonsynonymous, but no synonymous mutations—both cases are indicative of positive selection.

Table 2 also shows that the identified SNPs are not limited to certain regions of the genes. Some NLRs have SNPs in their C-terminus, other only in the NB-ARC domain or LRR domains, and in some cases SNPs are in all domains. Finally, we looked at the homology of our identified NLR with previously annotated NLRs from well-known pathosystems. As expected with a highly divergent gene family, only five NLRs show resemblance with previously verified NLRs. These are one homolog of R1A from potato, one of A*rabidopsis* RPP8, and three of *Arabidopsis* RPP13.

## Discussion

We annotated NLR genes in a wild tomato species and show proof of principle that pooled MiSeq sequence data (250 bp reads) can be used to infer population genetics statistics to determine variation of R-genes within one small population of *S. pennellii*. Moreover, we show that even in populations with reduced diversity, large numbers of polymorphisms are maintained in certain R-genes.

### Identification of NLRs

We predicted 220 NLR genes in *S. pennelli*, which is an improvement over the previous annotation, in which only individual domain occurrences had been described. This number is smaller than in cultivated tomato *S. lycopersicum* (326) and another wild relative *S. pimpenellifolium* (355) (Andolfo et al. 2014). Distribution among CNL and TNL classes is similar compared with both tomato species. Using current data, we find 93 NLRs (43%) to be putatively full length genes. In cultivated tomato this number is about 70%.

Aforementioned studies on tomato showed that so far only by manual curation and comparison with RENSeq sequence data one is able to identify all possible NLR-like regions on the genome. Unfortunately, this manual comparison will not allow accurate annotation of open reading frames (ORFs) and we consider it outside the scope of this artcle to perform and optimize such annotations. In this study, we therefore used the ORF as annotated by the *S. pennellii* genome project (Bolger, Scossa, et al. 2014). Reliance on these ORF could mean that not all NLRs in *S. pennellii* have been identified.

Indeed, our results indicate that in *S. pennellii* fewer NLRs are present. However, the phylogenetic reconstruction of the NLR family shows that our set of NLR genes covers the breath of NLR families observed in other *Solanum* spp. We are confident that we have not missed any known NLR family. For example, the manual curation in potato and tomato mainly revealed additional family members of known NLR gene clusters, only very few new singleton genes were identified. This curation mainly resulted in additional partial genes and increases the number of complete NLRs by 17% only, up to 221 complete NLRs in *S. lycopersicum* (Andolfo et al. 2014). Seeing that in *S. pennellii* we currently find only 93 complete NLRs, it is unlikely that after reannotation the number would be doubled. Therefore, we conclude that *S. pennellii* likely has lower numbers of NLRs than other sequenced tomato species and that the difference in NLR numbers could be caused by the habitat of *S. pennellii.* This habitat is relatively arid and one could assume there to be a lower pathogen pressure than for example in the habitat of *S. pimpenellifolium* or *S. lycopersicum* (Caicedo and Schaal 2004) which could cause higher rate of R-gene evolution (loss/gain).

### Successful Deep Sequencing using Few Resources

We showed that using RENSeq, we can cost and resource effectively, get a sufficient coverage over our target region using only 1/8th of an Illumina MiSeq lane. Kofler et al. (2012) suggested that for accurate pooled data processing very large numbers (>100) of individuals are needed to accurately capture all polymorphisms in the data set. They assume that in these cases on average each individual will be sequenced once or twice, with the high number of individuals making up for eventual bias due to sample preparation. This approach might be recommended for species where many individuals can be easily obtained like *Drosophila*, but is less feasible for larger species, or wild specimens, where collected samples might not contain that many individuals. We show that an alternative approach, using fewer samples, but assuring high coverage (on average >30 per diploid individual) can be as successful in identification of polymorphisms in a population. To assure the quality of the identified polymorphisms, we extensively tested four SNP calling packages and compared our data with selected genomic regions that were subjected to Sanger sequencing. The software Popoolation has been specifically designed for SNP calling in pooled samples of many individuals. We find that on our data set Popoolation (Kofler et al. 2011) slightly overestimates the number of SNPs present in the data, possibly due to lack of filtering options to remove biases in read composition introduced as an artifact of library preparation. GATK (McKenna et al. 2010) allows for more stringent filtering; however, no filtering thresholds could be identified so that GATK alone had the best result. This could be due to the nature of our data, which comes from enrichment sequencing and thus have very unequal coverage, big differences between introns and exons, and hence various biases that we could not fully capture with the available filters. Two other SNP callers significantly underperformed on our data, possibly because these were not optimized for pooled or mutliploid samples. In the end, we obtained the best results by merging the results and accepting only those SNPs that were called by both GATK and Popoolation. This strongly reduced the number of false positive calls, but might mean that in some low coverage regions minor alleles will not be counted. Validation using Sanger sequencing on selected regions showed however that in those regions 93.6% of all SNPs have been positively identified and also that only 3.6% of the SNPs were not identified in cases where they should have been. Overall, this shows that by combining callers, we are able to get both high sensitivity and high accuracy.

### Identification of SNPs in Samples with Reduced Diversity

Overall, we identified very low numbers of SNPs. This might be partly due to the stringency of the SNP calling; however, Sanger resequencing of a number of genes did not yield any additional polymorphisms. Many genes do not contain SNPs and no pattern can be observed in those that do. For example, SNPs are not predominantly found in either singletons or clustered genes. The likely explanation for this is the composition of the population. The sequenced plants come from a facultative selfing population collected in 1958 (Atico, Peru) and has been propagated during 5–10 rounds at the TGRC and Wageningen University as small populations of 8–12 plants (by pollen mixing and crossing). It is possible that the original population consisted of very few closely related specimens (maybe even one single plant) and that diversity has therefore been lost in the sampling and propagation processes. Our calculations show that the original proportion of genes with heterozygosity in the population could have been 10% or lower. With the current diversity found at 6%, this shows that even though the multiplication and initial sampling have decreased heterozygosity in our NLR genes, the initial population exhibited very low genetic diversity to start with. This is consistent with the diversity of self-compatible species to be much lower than that of self-incompatible species. This is exemplified by the fact that using AFLP markers more diversity (75% polymorphic sites) was observed within one accession of self-incompatible *S. peruvianum*, than between multiple accessions of self-compatible *Solanum* spp. like *S. pimpenellifolium* (7%) (Miller and Tanksley 1990). Recent studies confirm such high levels of polymorphisms to occur only in self-incompatible species (Städler et al. 2008).

We must note that the SFS will be strongly affected by genetic drift occurring during the multiplication process. This was seen in our global and per gene SFS with an excess of intermediate frequency variants. However, the genes we found to be polymorphic in our sample will have been diverse in the initial population due to possible past selective events and provide an insight in the number and location of polymorphisms in different genes.

### Maintained Polymorphism in C14 and NLR Genes

We can identify polymorphisms in our control gene, C14. C14 is a tomato protease targeted by multiple effectors from *P. infestans.* It has been shown to be under diversifying selection in wild potato (Kaschani et al. 2010). This does not seem to be the case in several wild tomato species (Shabab et al. 2008), which are thought not to be a natural host for *P. infestans*. Also in our population, C14 polymorphisms are predominantly synonymous and we detect no sign of diversifying selection. Interestingly, we did not identify any SNPs in another protease, Rcr3, which is under balancing selection in *S. peruvianum* (Hörger et al. 2012). Also, Pto, Fen, Rin4, Prf, and Pfi do not show polymorphism either, although they have been shown to be under selective pressure in *S. peruvianum* (Rose et al. 2007, 2011).

We identify after filtering 13 NLRs with one or more polymorphisms. Based on our above computations, we expect that heterozygosity at these genes reflects ancestral polymorphism in the initial population. These genes may thus show adaptation to different selective pressures which could be caused by the absence of or presence of certain pathogens on this specific population. Previous data from *Arabidopsis* suggests that when comparing different NLRs within a given genome, heterozygosity is larger in LRR regions (Mondragón-Palomino et al. 2002). However, we find no evidence that within one NLR polymorphisms between individuals are restricted to a certain region of the gene. This may be partly due to our current data set containing too few SNPs in too few genes to identify trends and link selection pressures on the genes to the place or domains where the selection occurs.

Five NLRs in our data set show a higher π*N* than πs, indicating possible positive selection. Due to the low diversity of our sampled population, we acknowledge that a high π*N*/πs ratio, however, does not necessarily suggest high positive selection pressure. As such, within-gene diversity could be a better indicator for evolutionary pressure in this population, because this could be a sign of balancing selection (Tellier et al. 2014). In terms of polymorphisms, certain individual genes indeed stand out. One of the genes that has maintained the highest number of polymorphisms within our population (Sopen11g028610) is an ortholog of *Arabidopsis* RPP13. RPP13 is known to maintain extreme high numbers of polymorphisms in wild populations (Rose et al. 2004), which is congruent with the highly polymorphic nature of its recognized effector Atr13 (Sohn et al. 2007; Rentel et al. 2008; Leonelli et al. 2011) and likely loss of fitness in the wild when one or multiple allelic variants disappear from the population. The highest number of polymorphisms can be found in Sopen12g022450. It has 83 putative SNPs, all in the LRR of the gene. It must be noted that this gene has been annotated as “partial” gene and might not be functional. As with the previous example, it would be interesting to know if Sopen12g022450 has a function in resistance and if its variants are maintained within different populations.

### Unraveling Short-Term NLR Evolution

A next step would be to test whether detected NLR variants show (partial) redundancies in terms of recognition. In grasses, a number of resistance genes from fast-evolving classes and classes with orthologs in 4 species have been cloned in rice and tested if they conferred resistance to 12 rice blast pathogen *Magnaporthe oryzae* strains. Fifteen out of 60 genes appear functional and no correlation was found between resistance and class or conservation between species (Yang et al. 2013). Resistances also appeared to be redundant between different pathogens, as observed in a larger study testing 132 NLR genes from cultivated rice. In the latter study, 43% of the R-genes confer resistance against on average 2.4 of the 12 isolates tested (Yang et al. 2013; Zhang et al. 2015). Recent studies have shown how several NLRs are required to work in pairs or networks, with closely related proteins sometimes conferring different functions (Eitas and Dangl 2010). Moreover, many NLRs seem to be highly expressed also in susceptible interactions and NLRs can even be contributed to quantitative resistance effects (Corwin et al. 2016). Thus, analysis of long-term evolutionary history using phylogeny would reveal only little about the recent selective pressures, state, and activity of the NLRs.

As plants and pathogens are thought to adapt to one another within and between populations, our method can be used to identify NLRs that are under acute evolutionary pressure (see also Rose et al. 2007; and theory in Tellier et al. 2014). This is illustrated here as the identification of *S. pennellii* genes that maintained polymorphisms in our low-diversity population, including an RPP13 homolog.

Another aspect of R-gene and resistance diversity could be found in potential CNVs for R-genes combined with rapid birth and death of new gene variants (Michelmore and Meyers 1998). The variation of binding affinity and kinetic properties of the RENSeq reaction do however make that the data could be biased toward certain sequences and hence do not allow to detect CNV within one population. However, when multiple populations would be sequenced simultaneously, the method could allow for copy number estimations and the identification of whole-gene presence/absence polymorphisms.

Follow-up work could thus include sequencing of multiple diverse populations, to help to identify functional R-genes from wild species, for example, NLRs under selective pressure or with CNV. This will help identify potentially important R-genes that can be used in disease-resistance breeding programs. These methods can in addition be compared with polymorphism data from wild pathogens, which will provide tests for current coevolutionary models (Tellier and Brown 2007; Tellier et al. 2014). To understand R-gene variation within and between populations of the same species, correlations with pathogen occurrence might help understand disease resistance ranges in crops and could solve questions on the molecular basis on nonhost resistance (Stam et al. 2014). It will help define the durability of certain resistance genes and will hence be beneficial for future resistance breeding programs.

## Supplementary Material

Supplementary figures S1–S3 and file S1–S6 are available at *Genome Biology and Evolution* online (http://www.gbe.oxfordjournals.org/).

Supplementary Data
