# Supplementary material for: Pooled Enrichment Sequencing Identifies Diversity and Evolutionary Pressures at NLR Resistance Genes within a Wild Tomato Population
Source: Genome Biol Evol. 2016 Apr 27;8(5):1501–15. doi: 10.1093/gbe/evw094 (PMC4898808; doi:10.1093/gbe/evw094)
Supplement: Supplementary Data [file supp_8_5_1501__index.html]

Pooled Enrichment Sequencing Identifies Diversity and Evolutionary Pressures at NLR Resistance Genes within a Wild Tomato Population — Supplementary Data 

# Pooled Enrichment Sequencing Identifies Diversity and Evolutionary Pressures at NLR Resistance Genes within a Wild Tomato Population

## Supplementary Data

files

- Supplementary Data - zip file
