# Supplementary figures and images for: Pooled Enrichment Sequencing Identifies Diversity and Evolutionary Pressures at NLR Resistance Genes within a Wild Tomato Population
Source: Genome Biol Evol. 2016 Apr 27;8(5):1501–15. doi: 10.1093/gbe/evw094 (PMC4898808; doi:10.1093/gbe/evw094)

S Fig 1

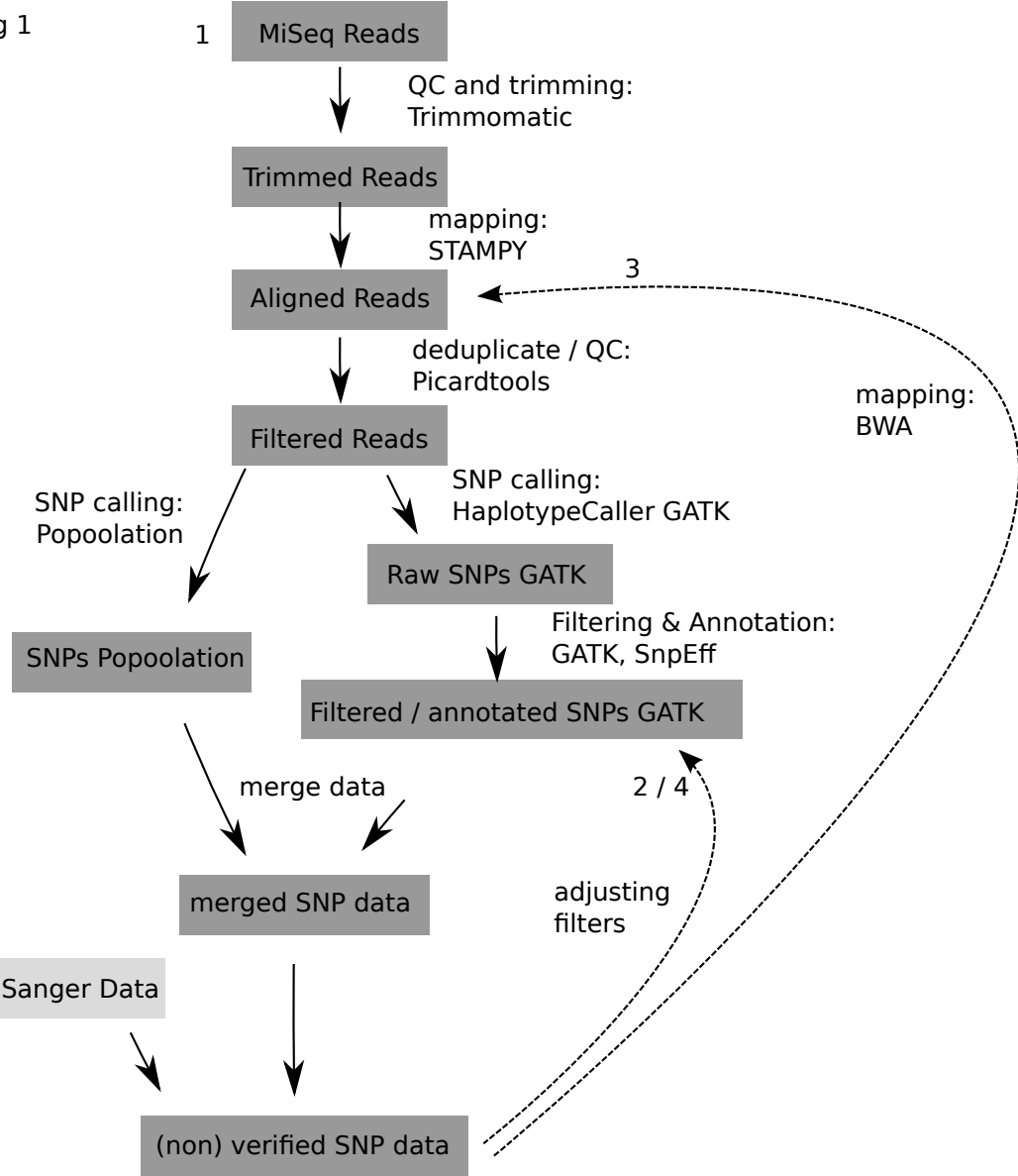

raw reads

run1

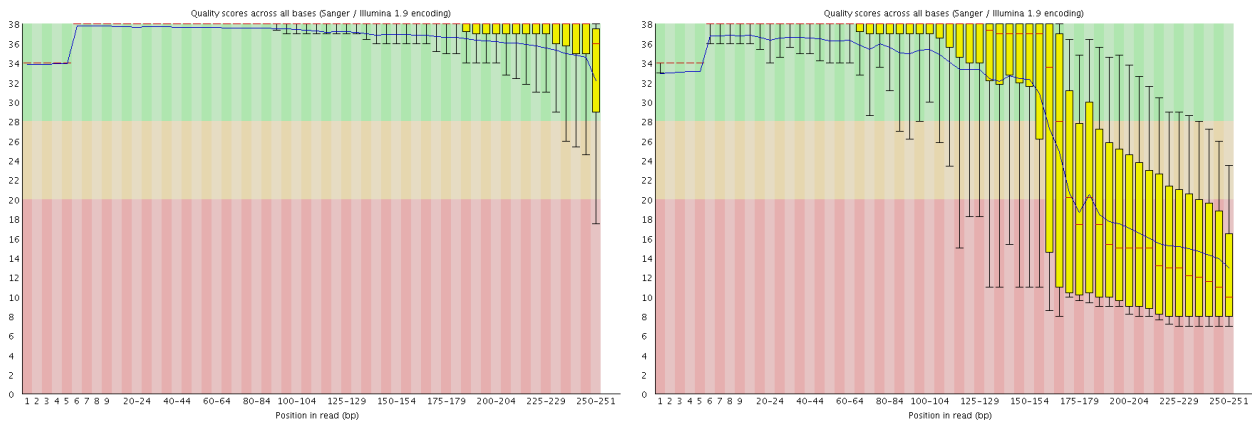

run2

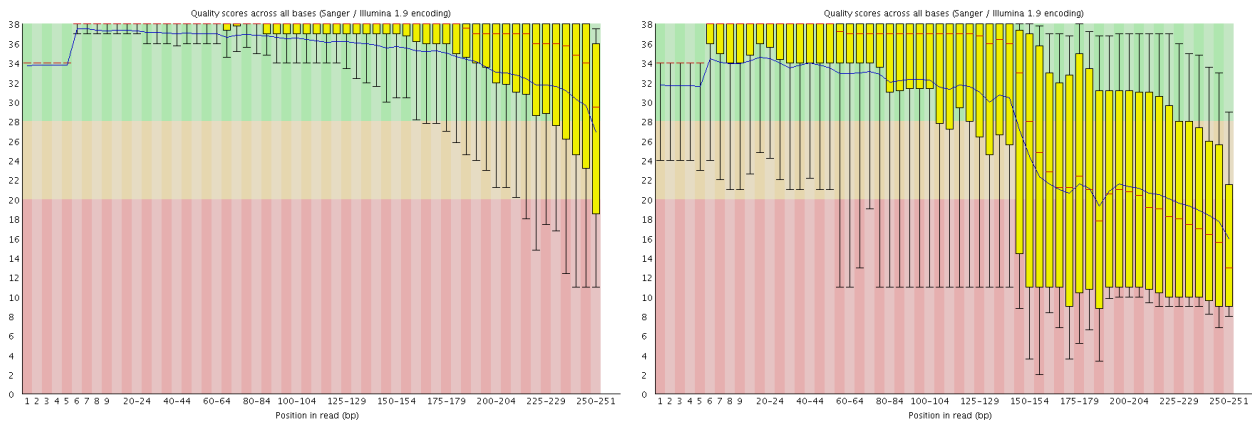

trimmed reads

run1

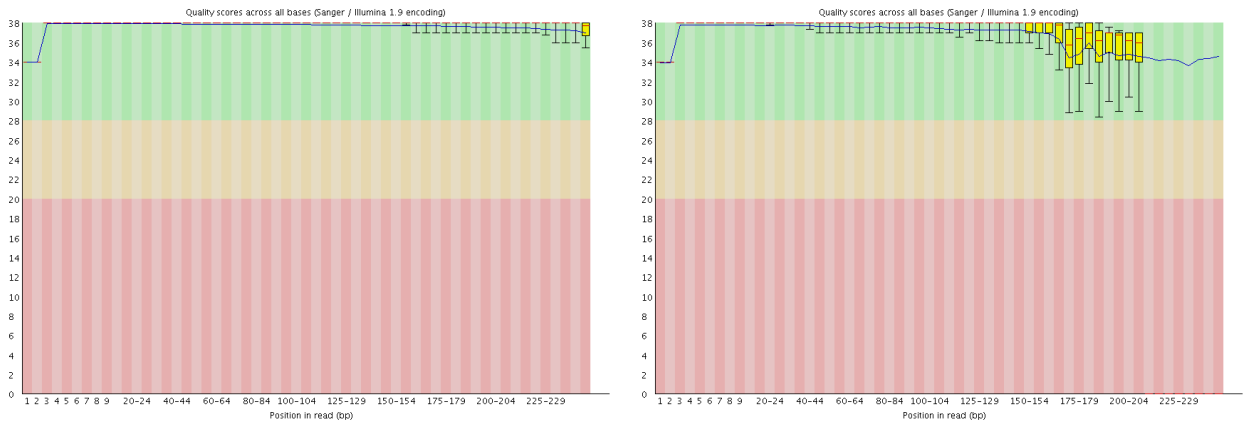

run2

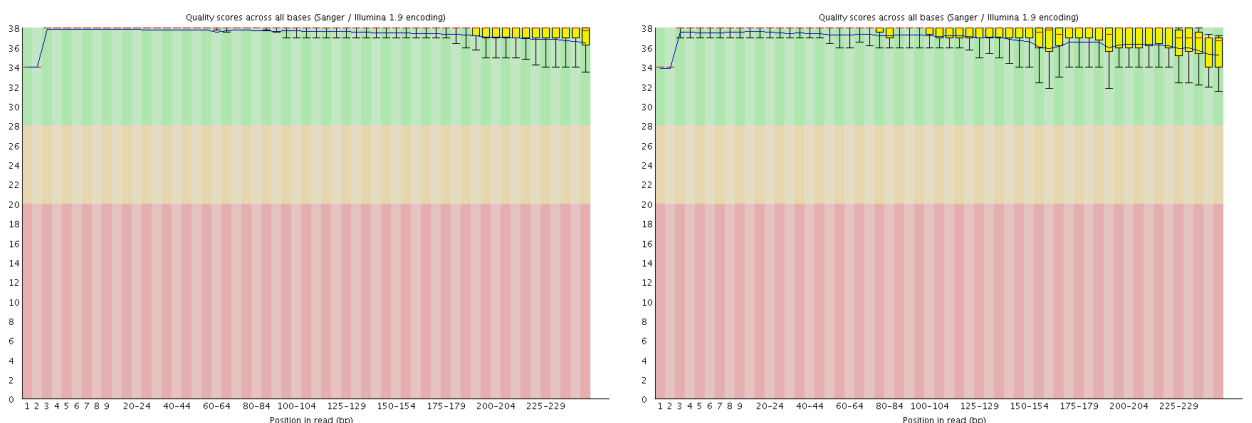

A

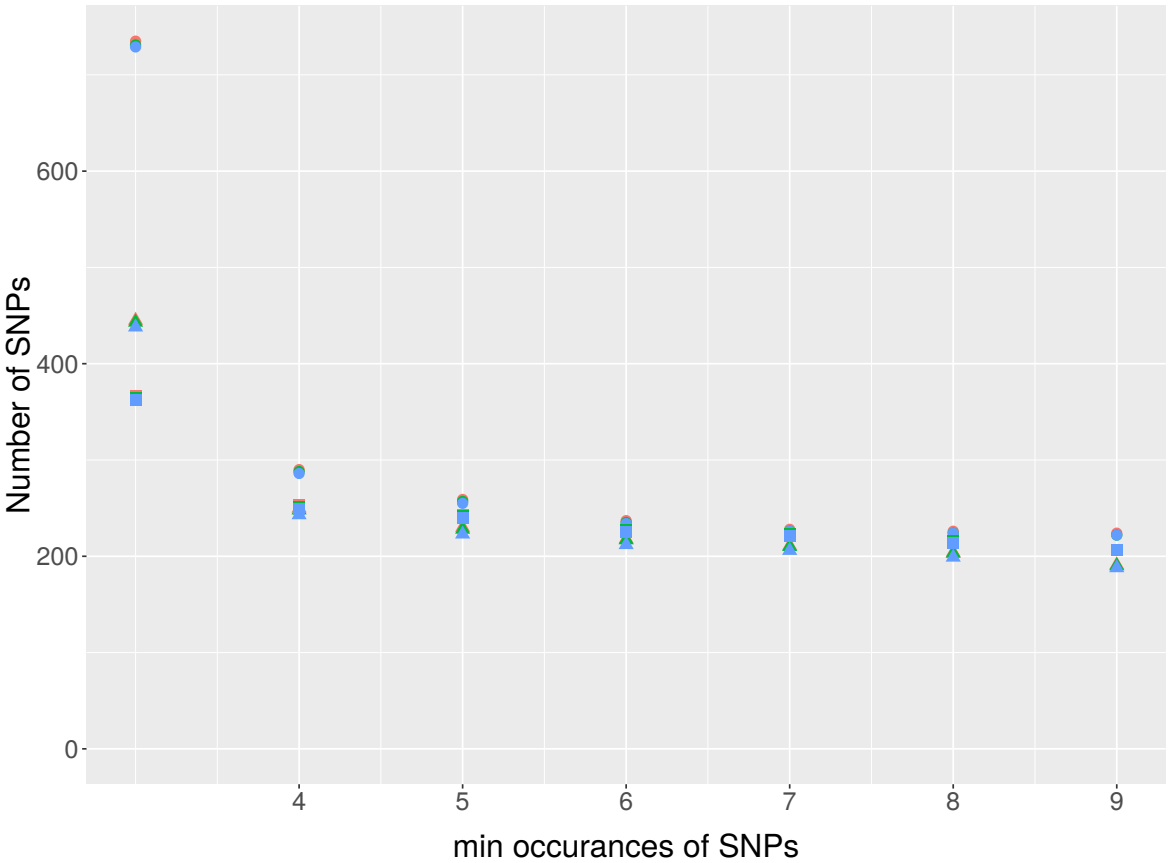

B

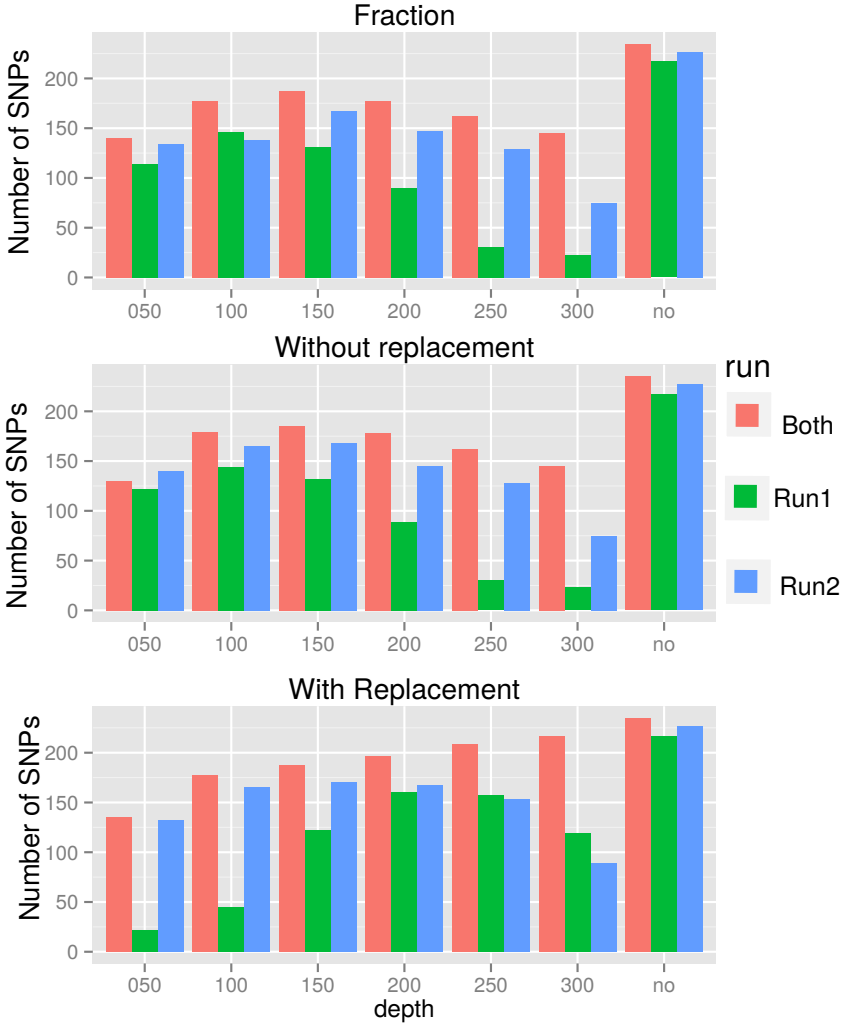

Supplement: Supplementary Data [file supp_evw094_suppl_data.zip › SupplementaryFigures.pdf]
